# Familiar story structures possess an evolutionary edge in memory

**DOI:** 10.1371/journal.pone.0341671

**Published:** 2026-03-03

**Authors:** Abla Alaoui-Soce, Diana I. Tamir

**Affiliations:** Department of Psychology, Princeton University, Princeton, New Jersey, United States of America; Xi'an University of Posts and Telecommunications, CHINA

## Abstract

Human beings demonstrate a universal impulse to share and consume stories. Over generations of transmission, within and across cultures, stories have evolved to develop regularities in their internal structures. Here, we investigated how two features of story structure – coherence and familiarity – impact recall as participants retold a story 5 days in a row. We predicted that familiar and coherent structures would be more stable over retellings. We measured stability using two novel story similarity measures of the (1) degree of structural change within storytellers, and (2) similarity in remembered structure across storytellers. Study 1 first validated our story similarity measure. Studies 2 and 3 then tracked the evolution of stories that varied in coherence and familiarity, respectively, using novel stories adapted from the popular “Cinderella” structure. Results showed that all stories became more structurally stable across retellings, with stories moving in a consistent direction (*i.e.*, towards a consistent final form). However, retellings of a story with a more coherent and familiar structure showed both greater stability *within* and similarity *across* minds than retellings of a story with an incoherent (Study 2) or unfamiliar structure (Study 3). Thus, using novel tools to measure story evolution, our findings suggest that familiarity and coherence of a story structure offered it an advantage in memory, both within and across minds.

## Familiar story structures possess an evolutionary edge in memory

Long before Disney took over the fairy tale market, stories like Cinderella, Snow White, and Sleeping Beauty had been part of the Western canon and beyond. One of the earliest known variants of Cinderella, “Rhodopis”, for instance, has been traced back to Greece in the first century B.C.E. [1]. Researchers in folklore and mythology have amassed catalogs of folktales in different societies across the world [2,3]. Their work reveals shared traits in stories *within* and *across* cultural boundaries, as stories are transmitted both through the generations and across cultures [2–5]. Some stories that still appear today, like the “Devil and the Smith” folktale, can trace their roots as far back as the Bronze Age, thousands of years ago [4]. However, not all stories are successfully transmitted or retain their structure. Stories undergo evolution: Some disappear entirely, others change drastically, and others still survive in more recognizable forms. What factors determine how stories evolve over retellings?

Stories do not evolve of their own accord. They evolve through people. To borrow Terry Pratchett’s metaphor, stories are “parasitic life form[s]” occupying the minds of people [6]. Story evolution relies on the minds that carry them as people pass them along with varying levels of accuracy. The evolution of story structure has taken place over the course of human history, and, as such, involves complex interactions between individuals and communities. Using novel story structure measures, we tracked an aspect of this evolution in real time—focusing on the cognitive determinants of this evolution. In other words, we observed how stories changed in a single person’s mind over several days, to understand why some stories survive better than others. Although we studied only a small set of stories here, we define story broadly: A story is any report of connected events involving behaving agents. From a toddler’s incoherent account of a broken cookie jar to the highly formulaic plotlines of weekly detective dramas, we consider all of these stories—albeit with varying degrees of structure.

### The role of schemas in story recall

The human mind is not a blank slate onto which a story is inscribed. Adult minds have expectations about how stories usually unfold. These expectations, or schemas, shape how people encode and recall new inputs [7–9]. In 1932, Bartlett first introduced the concept of schema [10]. In a series of seminal studies, Bartlett investigated the notion of memory as a reconstructive process by tracking how participants recalled narratives. In one study, when participants recalled a culturally unfamiliar Native American story, ‘War of the Ghosts’, they modified the story to fit their existing schemas [10]. They adjusted event sequences, added causal connections they felt were ‘missing’, and deleted elements that did not fit into their schemas. This pattern of distortion replicated across varying study designs, including individual recall (known as repeated reproduction) [11–13], recall in pairs [14], and recall in a chain [10], like the game of telephone. Bartlett’s research into schema and reconstructive memory has generated an impressive and active lineage of research [15].

Here, we build on this foundation to further test how schemas guide memory. We focus on two key factors that impact the extent to which a story fits into people’s schemas: Coherence and familiarity. We hypothesize that the more a story matches a person’s schemas, the more stable their recall.

### Coherence and familiarity are key elements of story structure

Coherence and familiarity are critical components of schemas. Schemas capture coherent sequences, in which the events follow a sensible, logical order that may be useful in predicting and navigating future situations [16,17]. As people are repeatedly exposed to similar experiences, they extract regularities across exposures, forming schemas that reflect these common patterns [17–19]. Over time, the more we encounter familiar structures, the more deeply ingrained these schemas become in our understanding of the world.

Past research offers strong evidence that coherence improves recall [20,21]. Coherent texts, both narrative [7,22,23] and expository [24,25], are easier to interpret and remember than incoherent ones [26]. Coherence is a multi-faceted construct: Temporal coherence refers to the logic of the temporal sequence between subsequent sentences. Referential coherence involves the repetition of key concepts or references across different parts of the narrative, helping readers connect information. Causal coherence refers to the causal connections between events, with causally related events remembered better [27,28]. Each type of coherence can independently improve memory, with causal coherence being especially effective [29,30].

In our studies, we disrupt all three aspects of coherence by presenting story events out of order, impairing the comprehensibility of the sequence. For example, if the events of a story were scrambled to read “They were on the train. They were awakened by an alarm. They got on the train”, the disruption of temporal, referential, and causal coherence would make it harder for readers to construct a coherent mental representation of the story. Such scrambling of texts has been associated with poorer memory performance [12,31]. Further, people tend to remember scrambled “scripted” actions (*e.g.*, going to a restaurant or to the dentist) by mentally reordering the events into their more typical, coherent sequence [9,32–34]. Essentially, participants work to restore a coherent version of the events, highlighting coherence’s strong influence on recall.

Familiarity is also integral to the notion of schemas. Schemas form through repeated exposure. Over multiple exposures, people come to understand the shared relationships and regularities in events that comprise a schema [17–19]. Once a person becomes familiar with a story schema, it influences their ability to comprehend, retain, and recall new and related stories [22,35]. Schemas are specific to a social and cultural context. People from different cultures remember stories differently – in a way that aligns with their familiar schemas [13]. For example, when asked to summarize a new story, people who summarized a story that followed culturally familiar story conventions produced more similar summaries than those who summarized an unfamiliar story [35]. Schemas are not fixed, however: Exposure to stories from other cultures can lead people to develop new schemas. As people from Western cultures are repeatedly exposed to unfamiliar stories, they show fewer distortions in recall [15].

In everyday stories, there is overlap between the constructs of coherence and familiarity. Familiar structures are typically more coherent and notions of coherence are developed through experience, as we familiarize ourselves with the statistics and causal relationships of the world [36]. Nevertheless, here we endeavor to manipulate coherence and familiarity independently. We expect that story structures that fit better with people’s schemas—whether because they are coherent or familiar—will be better retained.

### Story structure as event schemas

Over the past century, scholars have attempted to catalog and define story structures. Recent work has focused on defining story structure in terms of event structure [37]. This approach suggests that people segment continuous experiences into discrete units of activity, or events [37–39]. These event schemas are essentially canonical sequences of causally linked events that people apply to understand naturalistic experiences [39,40]. As mentioned above, schemas abstract away from single instances to encompass broader patterns of action. People are familiar with a multitude of such naturalistic schemas. For example, one might have a schema for dining out at a restaurant: Sitting down, ordering from the menu, waiting for the food, eating the food, paying the bill. One might have a finer grained schema for paying the bill: Flagging down the waitstaff, receiving the bill, pulling out a credit card, waiting for the processed bill, calculating the tip. Event schemas come into play when we engage with a story, influencing both their perception and recall [37].

We operationalized story structure by the sequence of events it comprises. The sequence of events is defined by both which events are included and the order of these events. We used a novel, automated tool–referred to as “story similarity” –to measure changes in story structure across retellings.

### Current research

How does a story structure’s coherence and familiarity determine its evolution in a person’s mind? To capture story evolution within the lab, we employ Bartlett’s (1932) narrative repeated reproduction design: In 3 studies, people read a story and then recall it across 5 subsequent days. By looking at transmission within a mind, our work, like Bartlett, can identify how schemas shape how stories are retained over time. We then build upon this foundation by employing novel, automated methods to quantify changes in story structure across recall. Prior work has often identified and measured distortions in recall by hand [11,14,15]. We capitalize on recent advances in natural language processing to develop a replicable automated process for systematically tracking the degree of change in story structure across retellings. We expect that this novel method will replicate prior work showing that schemas constrain recall, while also bringing new quantifiable insight into the degree of change across different story types.

In particular, we ask how two features of story structure – coherence and familiarity – impact recall. Study 1 first validates a new story similarity measure, in an exploratory test of how it tracks the evolution of a novel incoherent and unfamiliar story over time. We then use this story similarity measure to test a priori hypotheses about how the structure of stories would change across retellings of a coherent vs. incoherent and a familiar vs. unfamiliar story. In Study 2, we test the impact of coherence on the evolution of a story by comparing the evolution of a novel story that follows a familiar and coherent structure with the evolution of a familiar but incoherent version of the novel tale. We manipulate coherence by scrambling a familiarly-structured narrative. In Study 3, we test the impact of familiarity on the evolution of a story by comparing the evolution of that same familiarly structured story with that of an equally coherent, but unfamiliarly structured story. We manipulate familiarity by presenting two novel, equally coherent stories that include the same overall content, but differ in their underlying structure. The familiar story follows a familiar arc, modelled off of the highly familiar Cinderella tale. The Cinderella tale is a long enduring story, with surface level details varying across cultures, mediums and adaptations; over 300 versions have been documented [41]. We then measure the evolution of recall in two ways: (i) within an individual mind, replicating previous work on schematic influence [10–13], and (ii) across independent storytellers, to see if they converge upon a similar retelling. We expect familiar and coherent stories will be more stable in memory and converge to a greater extent across people.

## Study 1: Evolution of an incoherent and unfamiliar story

Study 1 has two aims. The first aim is methodological: To validate the new measure of story similarity developed to track how a story evolves across retellings. The second aim is to investigate the evolution of a story that lacks both coherence and familiarity. The story used in Study 1 was designed to read like fluent nonsense—a mishmash of randomly ordered events incompatible with participants’ prior knowledge. Participants should not be able to rely on existing schemas to facilitate encoding and recall. Our goal is to study how such a nonsensical yet fluent story evolves across retellings. Using a novel measure of story similarity, based on a representation of the event structure, we can track shifts in the story across retellings. With no familiar or coherent structure in the initial story to scaffold recall, the story would need to change significantly to better fit pre-existing schemas. Thus, we predict that the story will undergo large changes initially, and then change less across retellings as it becomes more stable and consistent with its final form.

### Methods

#### Participants.

Participants (*N* = 199) were recruited using Mechanical Turk (www.cloudresearch.com) to complete a 5 day study conducted between April 11^th^ and June 26^th^, 2019. We set a target sample size of 50, after attrition. Of the 199 participants who completed Day 1, 71 participants completed all 5 days of the task. Participants (*N* = 18) were excluded based on the following a priori exclusion criteria: (i) not meeting task requirements (*e.g.*, writing content unrelated to the story), (ii) writing insufficient text (*i.e*., word counts below 1 standard deviation from the mean; *M* = 126.50, *SD* = 48.53). These exclusions left us with a final sample size of 55.

All participants were U.S. residents fluent in English. Participants received a total of $3.50 for completing all days of the task (10 cents on Day 1, 20 cents on Day 2, 30 cents on Day 3, 40 cents on Day 4, and an additional $2.50 for completing all 5 days). Participants in this and all subsequent studies reported here provided informed consent in accordance with the Princeton University Institutional Review Board. Participant data (pre-exclusions and cleaning, as well as post-exclusions and cleaning) and analysis code for this and all subsequent studies can be accessed on OSF (https://osf.io/hu7ke/).

#### Procedure.

Participants completed the task over 5 consecutive days on Qualtrics (www.qualtrics.com). On Day 1, participants listened to a nonsense story that lasted 2 minutes and 22 seconds and consisted of 378 words (See *Supporting Information*). The story follows Sophia, a psychic, as she goes through a series of nonsensical adventures. Participants were asked to not take notes or replay the story to ensure they had no memory aids. After confirming they had listened to the entire story, participants were asked to recall the story as best they could. Participants were encouraged to write as much as possible.

On Days 2–5, participants were asked to recall the story from Day 1. Participants were invited to complete the task at the same time each day. They were allowed a maximum of 12 hours to complete the task. This protocol ensured that the time interval between retellings was no less than 12 hours and no more than 36 hours. By Day 5, participants produced 5 retellings of the initial story listened to on Day 1, one for each day.

Before data analyses, stories were cleaned in the following ways: (1) Spelling errors and typos were corrected. (2) Meta-commentary was removed. This included phrases like “if I remember correctly”, “I recall”, “I believe”, “I think”, as well as content about the difficulty of the task.

### Analyses

#### Story similarity measure.

We developed a novel story similarity measure to track how a story’s structure changes across retellings. This measure captures changes in the content and sequence of events in a story. The identification of events and event boundaries is a complex and ongoing line of research [42,43]. Here we use sentences as a proxy for events. While sentences do not perfectly map onto events, the length of the stories used in these studies allows us to generally ascribe one main action to each sentence.

To assess content changes, we measured the overlap in the events remembered in common across two retellings (*e.g.*, Day 1 vs. Day 3). To assess sequence changes, we measured how similarly ordered the remembered events are across retellings. Both the content and sequence aspects of the story similarity measure used Spacy’s semantic similarity calculator [44], which determines similarity by comparing word vectors, multi-dimensional representations of a word’s meaning. We then combined these two aspects—content and sequence—into a single overall story similarity measure. The following sections describe the steps involved in calculating the content, sequence, and overall story similarity measure.

#### Story content.

**Fig 1:**
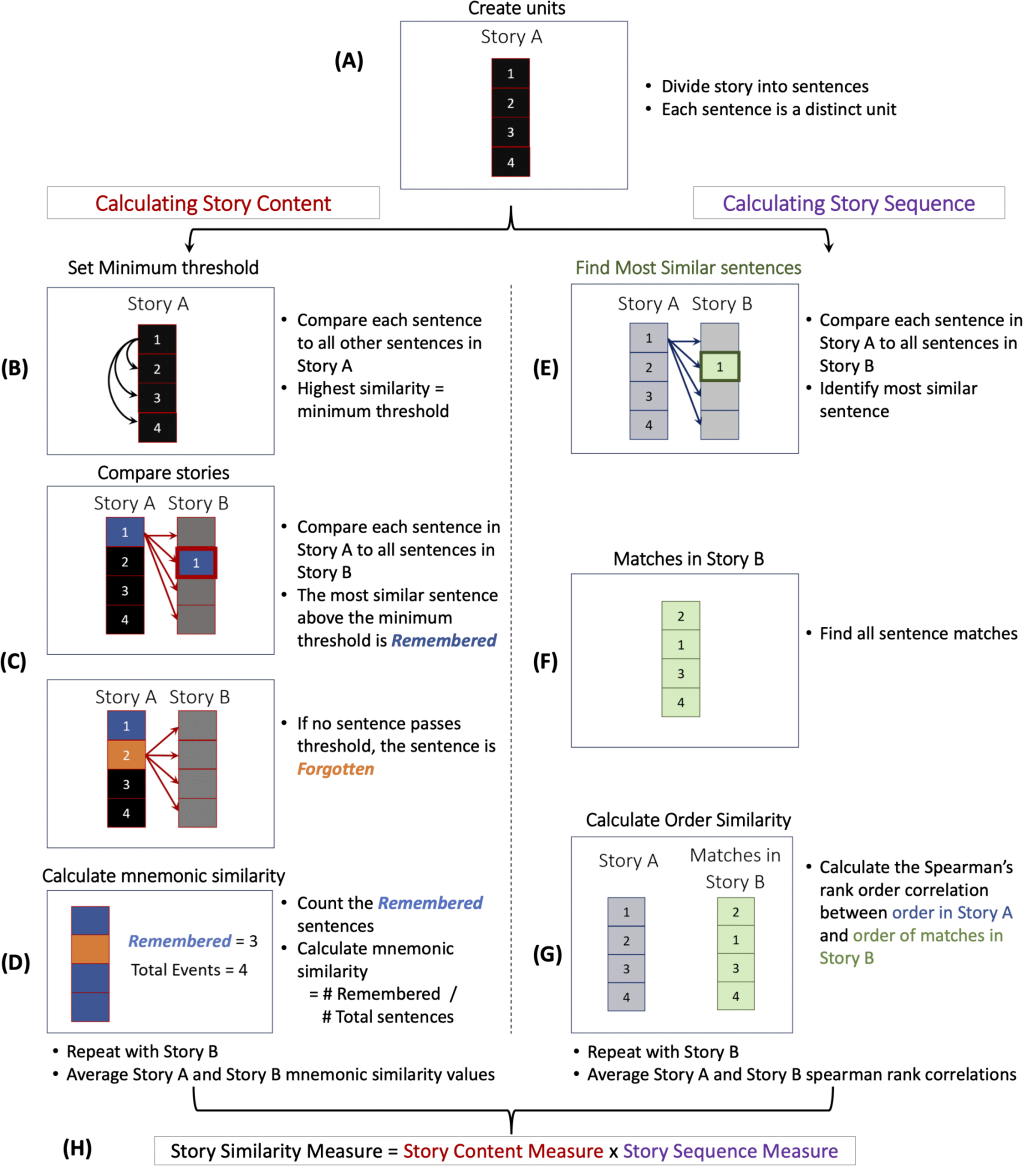
Story Similarity Measure. The similarity between two stories, Story A and Story B, is calculated using an automated measure of story content (A-D) and story sequence (E-G). The final story similarity value (H) is obtained by multiplying the content and sequence measures.

To capture how the content of a story evolves over retellings, we adapted the mnemonic similarity measure developed by Coman et al [45]. This measure tracks which events are remembered and forgotten in common between two retellings. This process proceeds as follows (Fig 1A-1D): First, we broke each story into its component sentences, which serve as a proxy for events in the narrative (Fig 1A). We then compared each sentence in Story A to every other sentence within Story A to determine the most similar sentence. The highest similarity value obtained from this comparison served as the minimum similarity threshold when comparing sentences across different retellings. This threshold allowed us to rule out sentences that likely did not represent the same events. The reason being: This highest similarity value represents the most similar a particular sentence X in Story A can be to another sentence Y in Story A. This other sentence Y describes a different occurrence. If the sentence in Story B that is most similar to sentence X in Story A is below this value, it likely does not depict the same occurrence. Therefore, we cannot qualify it as the same event.

Once we established this threshold, we compared each sentence in Story A to every sentence in Story B. If a sentence in Story B with the highest similarity value exceeded the minimum similarity threshold, we classified it as a *Remembered* event (Fig 1B-1C). If no sentence met this threshold, we classified it as a *Forgotten* event. This process was repeated for each sentence in Story A, yielding a list of events from Story A that are remembered and forgotten in Story B. Next, we calculated event content similarity by dividing the number of events remembered from Story A in Story B by the total number of events in Story A (Fig 1D).

This process was then repeated, now starting with Story B as the reference story, and comparing its sentences to those in Story A. The final content similarity measure was calculated by averaging the results from both comparisons (Fig 1D). This ensured symmetry in the way similarity was measured: Story A was just as similar to Story B as Story B was to Story A.

#### Story sequence.

To capture how the sequence of events in a story evolves over retellings, we developed a novel event order correlation measure (Fig 1A, Fig 1E-1G). Like the content measure, we began by dividing each retelling into sentences (Fig 1A). For each sentence in Story A, we used Spacy’s similarity measure to find the most similar sentence in Story B (Fig 1E). Unlike with the content measure, we did not apply a minimum similarity threshold when comparing sentences for sequence. The sentence with the highest similarity was assigned as a match. This ensured that the best possible match was selected for each sentence. This process was repeated for all the sentences in Story A (Fig 1F). A sentence in Story B could be matched to multiple sentences in Story A.

Once all sentences in Story A were matched with their most similar counterparts in Story B, we calculated the Spearman’s rank order correlation between the sequence of sentences in Story A and the order of their corresponding matches in Story B (Fig 1G). This correlation reflected how similarly the order of events is preserved across retellings.

Then, as with the content measure, this process was repeated, now starting with Story B as the reference and comparing its sentences to those in Story A. The final sequence similarity measure was calculated by averaging the two correlation coefficients (Fig 1G).

#### Story similarity.

Overall story similarity was derived by combining the content and sequence measures into a single score (Fig 1H). Specifically, we multiplied the content similarity measure by the sequence similarity measure (Story Content x Story Sequence). This combined measure captured how much of the story’s content is retained *and* how well the sequence of events is preserved across retellings. The resulting story similarity value ranges from −1 and 1, where higher values indicate greater structural similarity between two retellings. We used this measure to compare across retellings and between each retelling and the initial story. All pairwise t-tests were Bonferroni corrected for multiple comparisons and all Cohen’s d effect sizes were hedges corrected. This measure was used consistently for all studies reported.

#### Measuring the evolution of story similarity.

The story similarity measure allowed us to track how the structure of a story evolved across retellings. We captured this evolution by analyzing three forms of change: Stabilization, Consistency and Modification.

*Stabilization* is the process by which retellings increase in similarity over time. We measured this as the change in pairwise similarity between subsequent retellings from Day 1–5. We expected the similarity between Day 1 and Day 2 to be lower than the similarity between Day 2 and Day 3 or Day 3 and Day 4, as the story moved towards a more stable form with each retelling.

*Consistency* is defined as the extent to which retellings evolve in a consistent direction. We used Day 5, the last retelling, as the benchmark against which the earlier retellings are compared. We measured this as change in similarity to the final retelling (Day 5) from Days 1–4. We expected that, as participants continue to retell the story, the retellings would become more similar to the retelling produced on the final day, indicating consistency in the direction of story evolution.

*Modification* captures the degree of change undergone by the initial story. To measure this, we tracked the similarity between the initial story and each retelling (Days 1–5). We expected that stories with more familiar and coherent structures would undergo less modification across retellings, as they better align with people’s pre-existing schemas.

#### Validating the story similarity measure.

Before using the story similarity measure to capture evolution in our main analyses, we first validated it to ensure it was both informative and aligned with human judgements of similarity. To do so, we tested the extent to which our measure agreed with human ratings of story similarity, using the stories generated by participants in Study 1. The goal of our measure is to capture people’s *overall* judgment of similarity between two stories, which we argue is informed by both the content and sequence of events in the story.

To validate the measure, we recruited an independent set of participants (*N* = 212) from MTurk between September 11^th^ and 19^th^, 2020. Each participant rated the similarity of 5 pairs of stories on a scale from 1 (Extremely Different) to 7 (Extremely Similar). The pairs were selected to cover five specific scenarios: (i) the initial nonsense story paired with a participant-generated retelling, (ii) two retellings from the same participant from different days (*e.g.*, Participant X’s retellings on Days 2 and 4), (iii) two retellings from different participants on the same day of recall (*e.g.*, Day 3 retellings from Participants X and Y), and (iv) two pairs from different participants on different days of recall (*e.g.*, Participant X’s retelling from Day 3 and Participant Y’s from Day 5). This design ensured that we validated our automatic similarity measure using the full range of similarity measured in the study, including comparisons *within* participants (as needed for all three studies) and *across* participants (as needed for Studies 2 and 3). Each day of recall appeared at least once for each participant, and each pair of stories was rated by at least 3 different participants.

In total, 153 participants rated all the stories they were given, yielding at least 3 similarity ratings on 94 different pairs of stories. Intercoder reliability was moderate (ICC = 0.584), allowing us to average repeated similarity ratings to derive a single similarity rating for each pair of stories [46]. We then tested these human ratings against the outputs of our story automated similarity measure. We found significant correlations for all three components: (1) The content measure on its own, (*r*(92)=0.57, *p* < 0.001), (2) the sequence measure on its own, (*r*(92)=0.53, *p* < 0.001), and (3) the combined story similarity measure, (*r*(92)=0.67, *p* < 0.001). Importantly, the combined story similarity measure showed stronger alignment with human ratings of similarity than either the content measure (z = 2.11, *p* < 0.001) or sequence measure (z = 3.51, *p* < 0.001), confirming the import of both components for human judgments of similarity [47–49].

To ensure that the measure did not simply reflect differences in story length, beyond what a human rater might consider, we tested the correlation between the story similarity measure and human ratings while controlling for differences in word count and sentence count. We found similar correlations between our story similarity measure and human ratings of similarity when controlling (separately) for the effects of word count (*pr*(92)=0.67, *p* < 0.001) and sentence count differences (*pr*(92)=0.67, *p* < 0.001) [49]. Additional robustness checks further demonstrated that the story similarity measure did not simply reflect low-level story confounds, namely differences in story length (See *Supporting Information*).

### Results

This study asked how a story evolves in the *absence* of a coherent or familiar structure, using a story that does not follow a pre-existing schema to guide its encoding and recall. We tracked how a nonsense story changes across retellings. We expected that the story would start more unstable, undergoing significant changes in early retellings, and then become more stable in subsequent retellings. In addition, we expected that retellings would evolve in a consistent direction, becoming more similar to the last retelling produced on Day 5.

### Stabilization: Similarity across retellings

**Fig 2:**
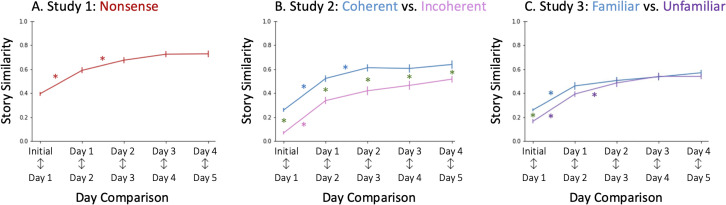
Stabilization: Similarity Across Retellings. Similarity increases across subsequent retellings in Studies 1-3, reflecting greater stability over time. Stability is higher for the Coherent, Familiar (blue) story. Color matched asterisks (e.g., red asterisks for Study 1) mark significant differences in story similarity between adjacent retellings. Green asterisks mark the significant differences between conditions. Error bars represent standard error.

To test if the story became more stable over retellings, we examined how similar each retelling was to each subsequent retelling (Fig 2A). We found a main effect of retelling on similarity (*F*(3.26,176.04)=62.681*, p* < 0.001, η^2^_*G*_=0.324), such that similarity was lowest between early retellings, and later stabilized into higher similarity values. Specifically, the similarity between the Initial story and Day 1 retelling (*M* = 0.39, *SD* = 0.12) was significantly lower than the similarities between all subsequent retellings [50]. Likewise, the similarity between Day 1 and Day 2 was lower than the similarity between all subsequent retellings*.* By Day 2, the story settled into a stable pattern, with similarity between Day 2 and Day 3 not differing from similarity between any subsequent retellings. These high similarity levels (around 0.7) suggested that the stories became highly stable by the second retellings, with only minor changes occurring across subsequent retellings.

### Consistency: Similarity to last retelling

**Fig 3:**
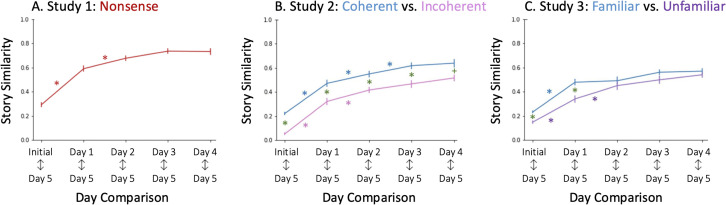
Consistency: Similarity to Last Retelling. Similarity to the final retelling increases across subsequent retellings in Studies 1-3, reflecting greater consistency over time. Consistency is higher for the Coherent, Familiar (blue) story. Color matched asterisks (e.g., red asterisks for Study 1) mark significant differences in story consistency between adjacent retellings. Green asterisks mark the significant differences between conditions. The green crosses mark marginal differences. Error bars represent standard error.

Next, to test if the story evolved in a consistent direction, we tracked similarity to the final retelling on Day 5 (Fig 3A). We found that the similarity of each story to Day 5 retelling increased over time, with a significant main effect of retelling on similarity (*F*(3.51,189.55)=114.028, *p* < 0.001, η^2^_*G*_=0.474). Similarity to the last retelling was lowest in earlier retellings before settling into consistently higher similarity. The similarity between the Initial story and Day 5 (*M* = 0.29, *SD* = 0.14) was significantly lower than all the similarities between each subsequent retelling and Day 5. The similarity between Day 1 and Day 5 was also lower than the similarity between each subsequent retelling and Day 5. By Day 2, however, we no longer saw differences in similarity to the last retelling. This suggests that by Day 2, the story has already settled into a relatively stable form consistent with the final retelling on Day 5.

### Modification: Similarity to initial story

**Fig 4:**
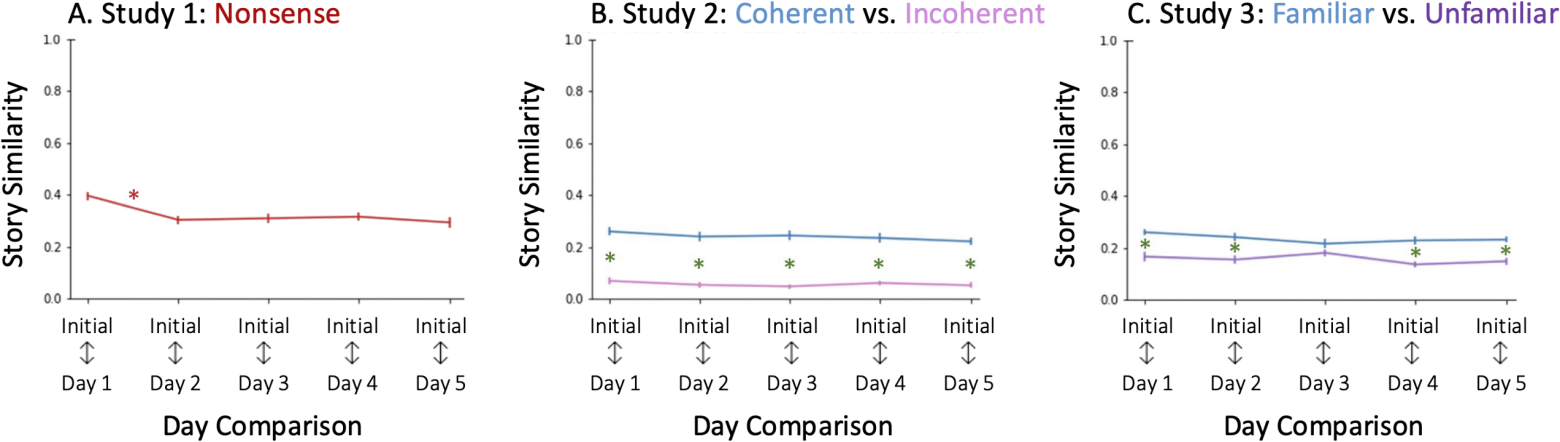
Modification: Similarity to Initial Story. Similarity to the initial story is generally stable after the first retelling. Modification is lower for the Coherent, Familiar retellings (blue), which remain more similar to the initial story than the Incoherent (pink) or Unfamiliar (purple) retellings. Color matched asterisks (e.g., red asterisks for Study 1) mark significant differences in story modification between adjacent retellings. Green asterisks mark the significant differences between conditions. Error bars represent standard error.

Lastly, we looked at how much the story was modified by measuring the similarity between the initial story and each retelling (Fig 4A). There was a main effect of retelling on similarity to the initial story (*F*(3.28,177.16)=13.762, *p* < 0.001, η^2^_*G*_=0.074). The story similarity between the Initial story and Day 1 retelling (*M* = 0.39, *SD* = 0.12) was significantly higher than the similarities between the Initial story and any of the subsequent retellings. However, the similarity between the initial story did not change across later retellings, suggesting the major structural modifications to the story occurred early, likely by the second retelling.

### Discussion

Study 1 tracked the evolution of an incoherent, unfamiliar nonsense story over five retellings and found that it reached high levels of stability as early as the second retelling. This suggests that participants rapidly modify the initial story into a form that is more stable in their recall. Most of the modifications to the initial story occurred during the first or second retelling, after which the story remained relatively unchanged. This does not mean that all subsequent retellings are identical, but rather that the core structure–both in terms of story content and sequence–remained highly stable from that point forward.

These findings highlight the utility of our automated similarity measure for tracking changes in stories across retellings. We first validated the measure by comparing it against human judgements of similarity, finding strong alignment between the two that cannot be explained by potentially superficial textual differences (*e.g.*, length). Then, by applying this measure to the progression of an incoherent, unfamiliar story, we established a baseline for how a story, with no initial structure or familiarity, evolves and stabilizes across retellings. This progression illustrates how stories might evolve when they begin without a pre-existing schematic structure.

Armed with this validated story similarity measure, we are now in a position to explore how different factors, such as coherence and familiarity, guide story evolution.

## Study 2: Evolution of a coherent vs. incoherent story

In Study 2, we test the influence of coherence on story recall. We manipulate coherence by comparing the evolution of an coherent story against that of an incoherent story. The coherent story is modeled off of the Cinderella-type tale, a tale for which we expect participants to have strong structural priors. The incoherent story was derived by scrambling the coherent story. We expect that coherence gives stories an evolutionary edge, such that they remain more stable, consistent, and less modified in memory. In addition to capturing evolution within a person, we also capture similarity *across* participants. We expect that the coherent story will be remembered more similarly across participants by the last day of recall.

### Methods

#### Participants.

Participants (*N* = 188) were recruited using Prolific (www.prolific.co) [51] between December 16^th^, 2019 and February 11^th^, 2020. As in Study 1, we set a target sample size of 50, after attrition. Of the participants who completed Day 1, 64 participants completed all 5 days of the task. Participants not lost to attrition were excluded for not meeting task requirements: For example, writing less than the required count, copy and pasting words over and over, or not writing the story at all, typing in different words. This left us with a final sample size of 56. Participants were randomly assigned to one of two conditions, the Coherent condition (*N* = 31) and Incoherent condition (*N* = 25). The different condition counts are due to different attrition and exclusion rates. Effects hold even when matching the number of participants across conditions (See *Supporting Information*).

All participants were U.S. residents fluent in English. Participants received a total of $7.60 for completing all days of the task ($2.20 on Day 1, 1.10 on Days 2–5 each, plus an additional $1.00 for completing all 5 days).

### Procedure

Participants completed the task over 5 consecutive days on Qualtrics. On Day 1, participants read and recalled one of two stories: 1) A Coherent story and 2) An Incoherent (scrambled) version of the same story (See *Supporting Information*). The Coherent story followed Sara, a high schooler who works to win a science fair that will earn her entrance into a university. The story was designed to match a well-established story schema. Specifically, this story was modeled after the main event structure of the Cinderella story, a folktale considered part of Western canon. The Incoherent story contained the same sentences as the coherent version, but these sentences were randomly shuffled to disrupt the continuity of the story. Each story was 758 words long. After reading the story, participants confirmed they read it completely. Next, participants recalled the story they had just read. Participants were encouraged to write as much as possible, with a minimum of 350 required to submit their response on each day.

On Days 2–5, participants were asked to recall the story from Day 1. Participants were invited to complete the task at the same time each day. They had 12 hours to participate. By Day 5, participants produced 5 retellings of the initial story, with first retelling on Day 1, and the last retelling on Day 5.

### Analyses

#### Story dispersion.

In Study 2, in addition to tracking changes *within* participants’ retellings (namely, stability, consistency and modification), we also compare stories *across* participants. We measured the similarity of all the participants’ stories at each day of recall, and measured their dispersion—the distribution of all recalled stories. Stories that are more similar cluster closer together, while stories that are more dissimilar are spread further apart. Dispersion was derived from calculations of similarity, using our story similarity measure, as follows:

First, we calculated the story similarity between all stories generated in Study 2, Study 3, and an additional study of retellings across participants (not reported here) based on the same initial stories used in Study 3 (These stories can be found on https://osf.io/hu7ke/). This included retellings excluded for length, and the initial stories given to participants. By comparing a large number of stories, we could more accurately map their relation to each other. These comparisons resulted in a similarity matrix of all the stories, which we converted into a distance matrix. Next, we applied Multidimensional Scaling to this distance matrix to map all stories in a 2-dimensional space. Within this space, we measured dispersion using Standard Distance Deviation: *SDD = sqrt(sum((x – mean[x])*^*2*^ *+ (y – mean[y])*^*2*^*)/ N(stories)),* where x and y are the coordinates of each story in the 2-dimensional space. We report the primary dispersion metric on the last day of recall, comparing dispersion for the Coherent vs. Incoherent stories. We used permutation tests to determine whether the difference between conditions was significant. The SDD for the remaining retellings, and comparisons across conditions are reported in the Supporting Information.

### Results

This study tested how coherence shapes story evolution. For each of our three metrics of evolution – Stability, Consistency and Modification – we first followed the evolution of each story type, the Coherent Story and the Incoherent Story, and then compared the two conditions to one another. In addition, we introduced the Story Dispersion measure, which employs our story similarity measure to derive similarity across participants at each day of recall.

### Stability: Similarity across retellings

To test whether the stories become more stable over retellings, we measured story similarity across subsequent retellings (Fig 2B). Across both stories (Coherent and Incoherent), we found a main effect of day [*F*(3.47,187.22)= 119.22, *p* < 0.001, η^2^_*G*_=0.476]. Stability increased over subsequent recalls for both the Coherent (*F*(3.01, 90.37)= 66.62, *p* < 0.001, η^2^_*G*_ = 0.431) and Incoherent story, (*F*(4, 96)= 53.54, *p* < 0.001, η^2^_*G*_=0.534). In both conditions, the similarity between the Initial story and first day of recall was significantly lower than similarity between all subsequent retellings. The story similarity of the Coherent and Incoherent stories settled quickly within the first two retellings*.*

We then tested our primary hypothesis, that the Coherent story would be more stable than the Incoherent story, by comparing stability across conditions. As expected, the Coherent story elicited consistently higher stability across all retellings than the Incoherent story [main effect of condition: *F(*1,54*)* = 26.056*, p* < 0.001, η^2^_*G*_ = 0.221]. This effect held when comparing stability on each individual day (See *Supporting Information* for day-specific results).

### Consistency: Similarity to last retelling

Next, we tested whether the stories evolve in a consistent direction (Fig 3B). To do this, we tracked the story similarity of retelling to the final retelling on Day 5. As expected, we found a main effect of day for both the Coherent and Incoherent story (*F*(3,162.14)= 166.36, *p* < 0.001, η^2^_*G*_ = 0.545): Consistency increased with retellings for both the Coherent (*F*(2.72,81.56)= 91.087, *p* < 0.001, η^2^_*G*_ = 0.493) and Incoherent (*F*(3.01,72.36)= 75.874, *p* < 0.001), η^2^_*G*_ = 0.617) condition. In both conditions, the similarity between the Initial story and Day 5, as well as the similarity between Day 1 and Day 5 were lower than all subsequent retellings’ similarity with Day 5. Importantly, for both the Coherent and Incoherent story, we never found that the similarity to the last retelling decreased. This suggests that the story was moving consistently towards a ‘final form’, rather than changing in inconsistent ways.

When then tested our primary hypothesis, comparing consistency across story conditions. Across all retellings, we found greater similarity to the last day of recall for the Coherent story than for the Incoherent story [main effect of story: *F(1,54)* = 22.48*, p* < 0.001, η^2^_*G*_ = 0.203], suggesting that the Coherent story remained consistently closer to its final form through all retellings. (This difference was marginal, *p* = 0.055, on Day 5.) (See *Supporting Information* for a table detailing the differences between story conditions.)

### Modification: Similarity to the initial story

Lastly, we tracked how the initial story is modified across retellings. To do this, we compared the story similarity of each retelling to the initial story (Fig 4B). For both the Coherent and Incoherent story, we did not find a main effect of day, suggesting that there were no differences in the similarity to the initial story across retellings. That is, independently for each story (Coherent and Incoherent), the initial story was equally preserved across retellings. (See *Supporting Information* for sensitivity power analyses [52,53].)

Next, we compared across story conditions to test our primary hypothesis, asking whether structure influences the degree to which the initial story is modified in memory. As expected, we saw greater similarity to the initial story for the Coherent story than the Incoherent story (main effect of story: *F*(1,54)=104.35, *p* < 0.001, η^2^_*G*_ = 0.554) across all days of retellings. That is, the initial Coherent story was better preserved in memory across retellings than the initial Incoherent story. (See *Supporting Information* for a table detailing the differences between story conditions on each day.)

### Story dispersion

**Fig 5:**
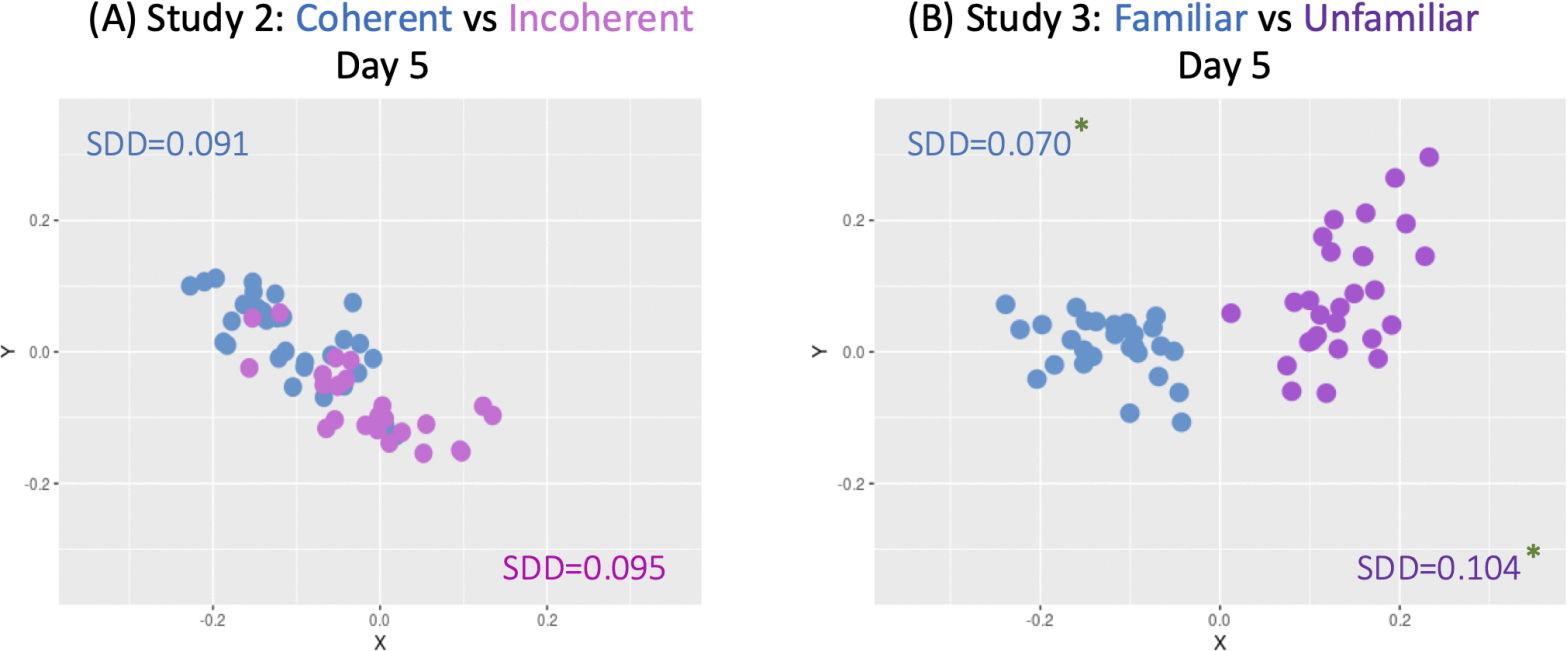
Story Dispersion. This figure illustrates the Story Dispersion results for Study 2 (A) and Study 3 (B) on Day 5. In Study 2, the Coherent retellings (blue) and Incoherent retellings (pink) are equally dispersed in space. In Study 3, the Familiar retellings (blue) is more tightly dispersed in space than the Unfamiliar retellings (purple). SDD above refers to the Standard Distance Deviation of each story grouping, color matched to its condition. Asterisks indicate that the condition SDDs are significantly different from one another.

So far, all the measures reported track changes within individual participants’ memory. These measures captured the first aspect of story survival. The second aspect of story survival has to do with story retellings across people. A story with an evolutionary edge should have greater agreement in recall across participants, such that the stories remain similarly structured across people. To test this, we measured story dispersion by the last day of recall (Fig 5A), and asked whether the Coherent story was more tightly dispersed than the Incoherent story. By Day 5, we did not find differences in dispersion for the Coherent (*SDD* = 0.091) versus the Incoherent (*SDD* = 0.095), *p* = ns. Against expectations, this suggests that participant recall was just as similar across those who recalled the Coherent story as those who recalled the Incoherent story. In addition, as shown in Fig 5A, there was clear overlap between the distribution of the Coherent story points (in blue) and Incoherent story points (in pink), suggesting that participants across both conditions converged upon a similar story structure.

### Similarity to the initial coherent story

**Fig 6:**
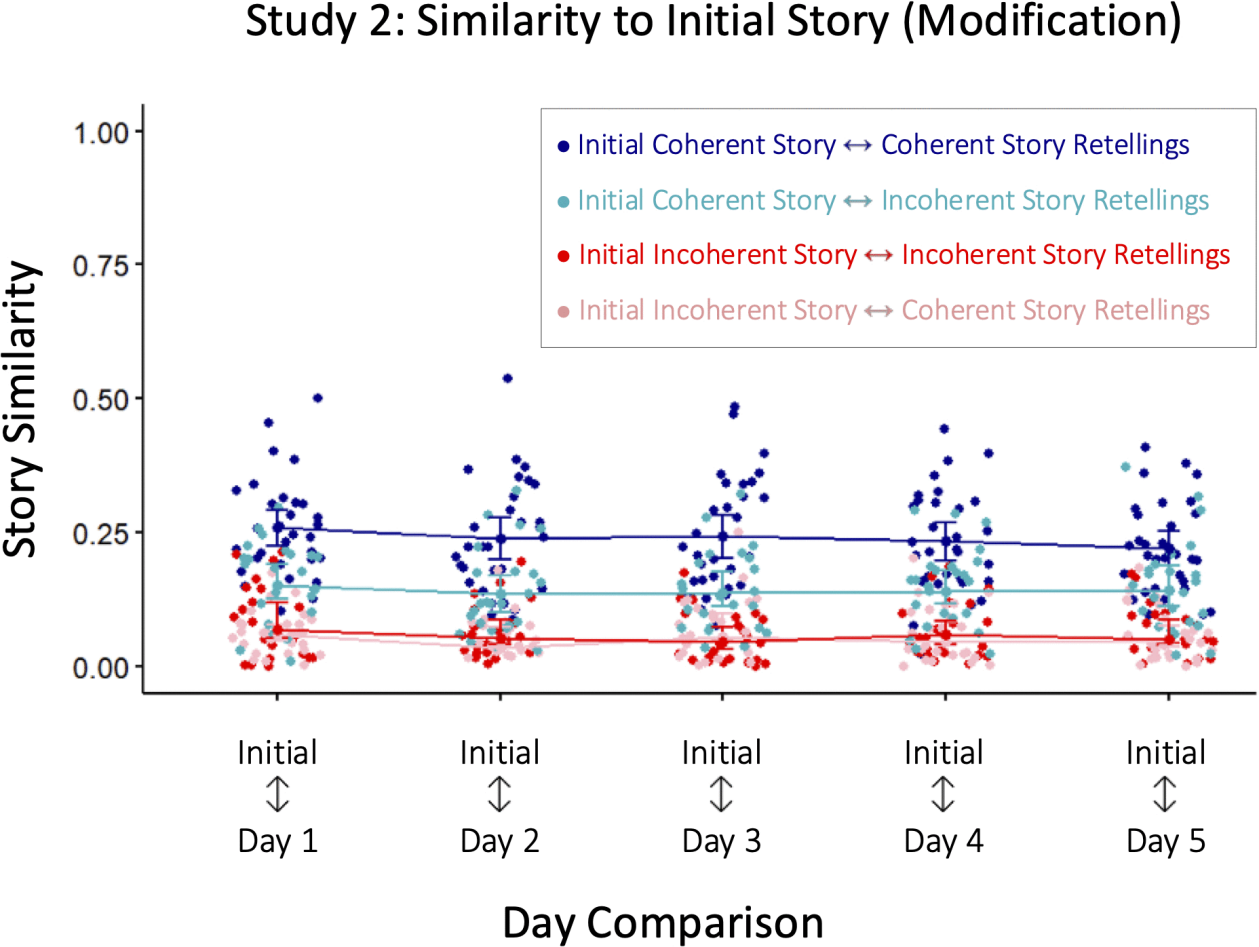
Modification. This shows the similarity of stories generated in the Coherent (dark blue) and Incoherent (light blue) conditions to the initial coherent story, and the similarity of stories in the Coherent (dark red) and Incoherent (light red) to the initial incoherent story. Both retellings were more similar to the initial coherent story than the incoherent story.

We conducted exploratory analyses to test one explanation for this convergence across conditions: That participants in the incoherent condition unscrambled the story to make it coherent, in particularly by bringing it closer to the initial story in the Coherent condition. If so, this could point to participants’ sensitivity to the structure represented by the Coherent story. We measured the story similarity of the retellings in the Incoherent condition to the initial Coherent vs. Incoherent story, at each day of recall (Fig 6). Results supported this interpretation: For all days, the Incoherent story retellings were more structurally similar to the initial Coherent story than they were to the initial Incoherent story (*F*(1,24)=33.64*, p* < 0.001). However, the Incoherent story retellings were less similar to the initial Coherent story than the Coherent retellings (*F*(1,54)=22.15, *p* < 0.001), suggesting that the Coherent story maintained an edge over the Incoherent story in retaining the Coherent story structure.

### Discussion

Study 2 tested the impact of structural coherence on story evolution. The results from Study 2 suggested that the Coherent story represents a more stable version of a story than the Incoherent story. Similarity across days of recall, similarity to the last retelling, and similarity to the initial story were higher for the coherent story. This provided evidence that stories with coherent structures allow for greater stability in memory. The Coherent story became stable very quickly, settling into a form that was more similar to its initial story. That is, the initial story was subject to less modification. The Incoherent story was less stable, and was subject to greater modification—as represented by the lower story similarity values.

Contrary to our hypothesis, we did not find differences in the dispersion of final stories across the two conditions. That is, cross-participant recall was just as similar–or as dispersed–for the Coherent story as for the Incoherent story. This suggests that the participants converged on a final story to a similar extent for both the Coherent and Incoherent story *and* that participants converged on a similar final story across both conditions. The Incoherent story is a scrambled version of the Coherent story, and participants seemed to unscramble the former into the latter. Across all days of recall, participants’ retellings of the Incoherent story were more similar to the initial Coherent story than to the initial Incoherent story that was their starting point. This unscrambling process occurred on the very first day of recall, and persisted across subsequent retellings. People may have unscrambled the Incoherent story, and in similar ways, because they were sensitive to its intended structure. This may be due to widespread familiarity with the Cinderella-form guiding interpretation and recall. We directly address the effect of structural familiarity on recall in Study 3 by comparing the evolution of this same coherent and highly familiarly-structured story to an equally coherent but unfamiliarly-structured story.

The story similarity values found here were lower than those found in Study 1. However, it is not meaningful to compare similarity values between studies for several reasons: Study 1 employs a significantly shorter story than Studies 2 and 3. The Study 1 story may thus be easier to remember because it constituted a much smaller memory load. In addition, Study 1 employed an audio recording of the story, as opposed to the written versions presented in Studies 2 and 3 which may have had an effect on people’s consolidation and recall of the initial story. Regardless of this difference across studies, however, the trends *within* studies do give insight into changes across retellings. Study 1 and Study 2 both demonstrate how stories become more stable in memory over days of recall.

## Study 3: Evolution of a familiar vs unfamiliar story

In Study 3, we test the influence of structural familiarity on story recall. We manipulate familiarity by comparing the evolution of a story with a familiar structure (referred to as the Familiar story) against that of a story with an unfamiliar structure (referred to as the Unfamiliar story). The Familiar story follows the structure of the Cinderella tale (the same story used in Study 2), the Unfamiliar story follows an unfamiliar structure, modified to be different from the Cinderella-type sequence of events. Both stories are new to participants. We expect that structural familiarity gives stories an evolutionary edge, such that the familiar story will be more stable and consistent, and subject to less modification in memory. As in Study 2, we also capture similarity *across* participants. We expect that the familiar story will be remembered more similarly across participants by the last day of recall.

### Methods

#### Participants.

As with Study 2, participants (*N* = 128) were recruited using Prolific. Based on Study 2’s results, we determined that a minimum sample size of *N* = 46 would be necessary to achieve 95% power to detect a main effect of story condition on Stability. We set a target sample size of 50. Participants were recruited between February 10^th^ and February 21^st^, 2020. Of the participants who completed Day 1, 55 usable participants completed all 5 days of the task. Participants were excluded using the same exclusionary criteria as in previous studies. Participants were randomly assigned to one of two conditions, the Familiar condition (*N* = 27) and Unfamiliar condition (*N* = 28).

All participants were U.S. residents fluent in English. Participants received a total of $7.60 for completing all days of the task, following the same payment scheme as in Study 2.

### Procedure

As in Study 1 and 2, participants completed the task over 5 consecutive days. The procedure matched that of Study 2, with one major change in manipulation. Participants either read the Familiar Story (Word Count = 758), the same story modeled off of the Cinderella-type story as used in Study 2, or an Unfamiliar story (Word Count = 756). The Unfamiliar story was written to include many events similar to those in the Familiar story, and to match as closely as possible for length, reading ease (Flesch Reading Ease for Familiar Story = 72.297, for Unfamiliar Story = 72.246) and cohesion (Deep cohesion percentile for Familiar Story = 29.81, for Unfamiliar Story = 26.76) according to Coh-Metrix measures [54]. It was made unfamiliar by changing Sara’s role from that of science fair participant to that of a science fair organizer. Instead of the story following Sara’s rags to riches tale, she earns a college scholarship within the first few lines. The focus of the story is primarily on Sara’s journey to help someone else from her old neighborhood be recognized for their project. (See *Supporting Information* for the story.) We expected that this story would thus no longer easily map onto any existing popular story type.

As in Study 1 and 2, by the end of Study 3, participants who stayed throughout produced 5 retellings of the story they read on the first day (Days 1–5).

### Analysis

Study 3 applied the same analysis techniques used in Study 2 to track the structural changes of the stories across days of retellings and story agreement across participants (story dispersion).

### Results

Study 3 asks how structural familiarity shapes the evolution of stories: We first tracked the evolution of each story type (Familiar and Unfamiliar) and compared these evolutions to one another. We then look at story dispersion, which captures agreement across participants.

### Stability: Similarity across retellings

To test whether story similarity increases across retellings, we looked at stability across days of recall (main effect of day: *F*(4,212)=119.26, *p* < 0.001, η^2^_*G*_ = 0.452), (Fig 2C). For both the Familiar (*F*(4,104)=48.040, *p* < 0.001, η^2^_*G*_ = 0.406) and Unfamiliar story (*F*(2.93,79.09)=72.837, *p* < 0.001, η^2^_*G*_ = 0.496), we found that stability typically increased across retellings. As expected, for both conditions, the similarity between the Initial Story and Day 1 was lower than the similarity between all subsequent retellings. In the Unfamiliar condition, the similarity between Day 1 and Day 2 was lower than the similarities between all subsequent retellings. For the following retellings of both conditions (Day 2 with Day 3 onwards), there were no significant differences between similarity across days. This pattern of evolution suggests that the Unfamiliar story was also evolving to become more stable in memory.

Next, we tested our primary hypothesis, asking whether the Familiar was more stable than the Unfamiliar story: We did not find a main effect of story. (See *Supporting Information* for sensitivity power analyses [52,53].) However, as expected, we found a difference in stability in the first day of retellings. Similarity between the Initial story and Day 1 was higher for the Familiar story than for the Unfamiliar story (*F*(1,53)=20.820, *p* < 0001, η^2^_*G*_ = 0.282). (Fig 2C). For subsequent retellings, there were no differences between the two story lineages. In other words, the Unfamiliar story started out less stable in participants’ memory. However, as early as Day 2, the Unfamiliar story closed the gap, matching the Familiar Story in terms of stability across retellings. Indeed, there was a marginal interaction effect between story and day of retelling (*F(4,212*)=2.37, *p* = 0.054, η^2^_*G*_ = 0.016) (See *Supporting Information*).

### Consistency: Similarity to last retelling

Next, we tested whether stories were changing in a consistent direction by tracking changes in similarity to Day 5 (Fig 3C). For both the Familiar (*F*(4,104)=68.30, *p* < 0.001, η^2^_*G*_ = 0.476) and Unfamiliar story (*F*(3.05,82.43)=82.43, *p* < 0.001, η^2^_*G*_ = 0.468), the retellings consistently evolved toward their final form. For both conditions, the similarity between the Initial Story and Day 5 was, once again, lower than all subsequent retellings’ similarity to Day 5. For the Unfamiliar condition, the similarity between Day 1 and Day 5 was also lower than all subsequent retellings’. By Day 3, however, both stories settled into a high similarity to Day 5 with no significant differences as compared to later retellings’ similarity to Day 5.

For both the Familiar and Unfamiliar story, similarity to the final form never decreased across retellings. This suggests (as in Study 1 and Study 2) that, as the stories were told and retold, they changed in a consistent direction (main effect of day: *F*(3.37,178.68)=139.624, *p* < 0.001, η^2^_*G*_ = 0.465).

Next, we tested our primary hypothesis, and looked at whether the Familiar story was more consistent than the Unfamiliar story. We found a main effect of story, driven by the earlier retellings (*F*(1,53)=5.12, *p* = 0.028, η^2^_*G*_ = 0.061). That is, the differences between stories only held for earlier retellings: The Initial story (*F*(1,53)=25.16, *p* < 0.001, η^2^_*G*_ = 0.322, 95% CI [0.05, 0.12]) and Day 1 (*F*(1,53)=10.97, *p* = 0.001, η^2^_*G*_ = 0.171, 95% CI [0.05, 0.22]; Fig 3C) retellings were more similar to the last day of recall for the Familiar story than the Unfamiliar story. For subsequent retellings’ similarity to Day 5, there were no differences between story conditions. Once again, the Unfamiliar story closed the gap with the Familiar story. This was highlighted by an interaction effect between story and day of recall (*F(3.37,178.68*)=3.17, *p* = 0.027, η^2^_*G*_ = 0.018). (See *Supporting Information*.)

### Modification: Similarity to initial story

Last amongst the within participant measures, we looked at how the initial story is modified across retellings (Fig 4C). As in Study 2, we did not find a main effect of comparison between days, suggesting that there were no differences in the similarity to the initial story across retellings. (See *Supporting Information* for sensitivity power analyses [52,53].)

However, we did, as in Study 2, find a main effect of story (*F*(1,53)=23.83, *p* < 0.001, η^2^_*G*_ = 0.212), with the Familiar story (*M* = 0.23, *SD* = 0.02) being more similar to the initial story than was the Unfamiliar story (*M* = 0.16, *SD* = 0.02). This effect was found for all days of recall, except for Day 3 (See *Supporting Information* for pairwise differences.) These differences suggest that, as the story was told and retold, the Unfamiliar story was subject to greater modification than the Familiar story. (See *Supporting Information*.)

### Story dispersion

Finally, we compared how stories are told across participants using the story dispersion measure. We looked at the dispersion of stories between participants on the last day of recall. We found a significant difference in dispersion, with the Familiar story having lower dispersion (*SDD* = 0.070) than the Unfamiliar Story (*SDD* = 0.104), *p* = 0.046 by Day 5 (Fig 5B). These differences started to appear by Day 4 (See *Supporting Information*). This suggests that recall was more similar across participants for the Familiar story than the Unfamiliar story. In other words, the Unfamiliar story recall diverged more across participants.

Unlike in Study 2, we found no overlap in the distribution of the Familiar story points (in blue) and Unfamiliar story points (in purple). The two groups of stories occupy distinct parts of the 2-d story space we generated (Fig 5B). This suggests that, as the stories were told and retold, they did, indeed, remain two distinct story lineages.

### Discussion

In Study 3, we asked how stories with varying degrees of structural familiarity fare within an individual’s memory. We found that structural familiarity allows for greater initial structural stability. The structurally familiar story started out more stable in participants’ memory. This suggests that a familiarly structured story, as one modeled after the Cinderella-type tale, latches better onto memory. The familiarly structured story does not require as much modification in order to become stable in participants’ memory. The Unfamiliar story, on the other hand, was modified more in individuals’ memory, as demonstrated by lower levels of stability in earlier retellings. However, across retellings, the unfamiliar story becomes more stable, suggesting that these modifications lead the story toward more stable forms in people’s memory. Indeed, participants’ memory processes seem to rapidly and efficiently modify the unfamiliarly structured story into a more stable form: As early as Day 2, there is no longer any difference between the stability of the Familiar and Unfamiliar story. While this is not addressed in this series of studies, future research could investigate the kinds of modifications that allow for greater stability. What changes occurred in participants’ memory that allowed the unfamiliar story to become more stable across retellings?

Participants differed in how they modified the Familiar and Unfamiliar stories. There was a greater divergence in recall for the unfamiliarly structured story than for the more familiarly structured story. By the last day of recall, the Unfamiliar story was more dispersed in story space than the Familiar story. The familiarly structured story, in contrast, was recalled with greater agreement across participants, pointing at an evolutionary edge: Not only is it subject to less modification, it is also recalled more faithfully across people. A Cinderella tale remains a Cinderella tale no matter who recounts it.

## General discussion

Stories have developed, over history, into a consistent set of shapes that define a culture’s modern narrative repertoire. How do stories evolve and take shape over retellings? Across 3 studies, we start to address the cognitive component of this question by varying the initial coherence (coherent or incoherent) and familiarity (familiar or unfamiliar) of stories. We ask: How do these story shapes guide people’s memory for novel stories? In these studies, we used cultural commonness as a proxy for familiarity, selecting the Cinderella structure as an extremely common and, therefore, highly familiar story structure. As expected, we find that a coherently and familiarly structured story is more stable and subject to less modification in people’s memory. An incoherently or unfamiliarly structured story, on the other hand, undergoes greater modification in people’s memory before becoming comparably stable.

Crucially, our results also show that people converge in how they recall a familiarly structured story. By the fifth day of recall, the stories generated by participants are more structurally similar to each other when the initial story was familiarly structured than when it was unfamiliarly structured. This result builds upon prior findings to show that, not only is recall for a familiarly structured story more stable *within* people [9,13], it is also more consistent *across* people. Familiar stories fit better into *individuals’* schemas, *and* these individual schemas are shared across people. People’s recollections for familiarly structured stories converge, whereas the greater modifications across retellings cause people’s recollections for unfamiliarly structured stories to diverge. The studies reported here cannot provide insight into exactly what these modifications are. However, previous research [9,22] suggests that unfamiliar stories are modified to better fit people’s schemas. To the extent that recall accuracy reflects schematic fit, the current results likewise suggest that stories are modified over retellings to better fit into people’s schemas. These schemas, however, may not be as consistent across people as those that align with familiarly structured stories: Study 3 finds that these modifications lead to greater differences in recall for an unfamiliarly structured story, suggesting that, without the shared Cinderella structure, recall may be guided by different schemas across people. This is in contrast to Study 2, where a scrambled familiar story is reordered in retellings to partially restore the intended structure, such that retellings of the scrambled story overlap with those of the initial (coherent) story.

Measuring story similarity across people allows us to tap into existing shared cultural schemas. Our studies are *not* designed to develop a shared culture in a lab setting, as has been explored in social transmission and cultural evolution studies [55–57]. These studies have primarily investigated schemas in the context of cumulative cultural evolution, typically understood as social learning involving the accumulation of helpful modifications over time [56–60]. For example, a group of participants may each transmit a story to one other participant. Researchers can then identify how the story has changed by the end of the chain to learn about the mechanisms of cultural development and evolution. This method has revealed a strong tendency for groups to create structure. For example, as people transmit artificial languages in chains, the languages become increasingly structured [57]. When transmitting descriptions of everyday events, people tend to maintain higher level structural information, and lose the low-level details [55]. Thus, this work allowed researchers to track the micro-development of cultures, *i.e.,* schemas shared across minds, within a lab setting by tracking transmission across a group of minds.

At no point in our studies are individuals transmitting their retellings to other individuals. Rather, we are interested in how individuals’ schemas manifest as they tell a story again and again. If people have existing shared cultural schemas, we should see agreement in their retellings–*without* any communication whatsoever. Greater agreement suggests that individuals independently converge to story structures in a manner constrained by their familiarity with those structures. In other words, similarity across individuals (acting independently of one another) reveals a shared culture.

We argue that familiar story structures serve as a kind of shared cultural schema, such that people within a cultural context share similar story structures. These shared story schemas evoke the notion of ‘cultural attractors’ [61], reflecting a collective cognitive alignment that may stem from culturally or universally determined *pre*-existing cognitive biases [62,63]. We cannot know from the current investigation the extent to which familiar story structures are ‘destined’ to exist regardless of cultural context. We do, however, propose this work within the lineage of schema research [9,18], and as such suggest that the development of story schemas comes from a lifetime of experience, where the interaction between people’s schemas and stories can be understood as a mutually reinforcing feedback loop: Commonly shared story types contribute to the development of internalized story schemas; this leads to both new schematic stories being better retained and non-schematic stories being modified to fit existing schemas; which, in turn, leads to more schematic stories being more commonly shared. Of course, differences in individuals’ experiences with stories will certainly lead to differences in their story schemas, but there is overlap–a shared story canon. It remains an open question why *these* are the story structures that entered the canon. In other words, why are these the stories that became familiar? Perhaps it is simply that these are the stories that have been told over and over again, passed down through the generations. Alternatively, it is possible that there are characteristics of certain story structures that make them better stories, above and beyond mere familiarity—in which case the argument could be made for certain story structures serving as ‘cultural attractors.’.

### Limitations and future research

The work reported here explores how familiar story structures influence memory. Familiar story structures may activate schemas shared by adults in a similar cultural setting, and thus adults converge and stabilize in their retellings of the story over time. However, this conclusion is limited by our use of a single story structure: Cinderella. This story structure was chosen because it is an extremely widespread and active story structure, as evidenced by a consistently high Google Ngram mentions [64], even relative to other popular narratives (See *Supporting Information*). We welcome future research to test the extent to which our conclusions apply beyond this particular story structure. In particular, future work would need to test the extent to which our effects could be explained by other key features of a story. For example, people may remember stories more accurately the more enjoyable, well-written, easy to understand, entertaining, or informative they are. It is worth noting, however, that it may be impossible to completely disambiguate the effects of story features from the effects of familiarity, or schema strength. Familiar story structures may share certain story features that promote their success, both at the individual and cultural level. We look forward to clarity on the extent to which these features arise from or explain an effect of schema familiarity on memory.

The studies reported here assume that our novel story activated the Cinderella story schemas. The story was designed to have the same underlying structure as Cinderella but at the same time, share little to no content. It is difficult to determine if the story presented to participants reliably activated the corresponding schema. What does it mean to activate the corresponding schema? Research suggests that event schemas are hierarchical, with multiple levels of specificity. During narrative or naturalistic perception, multiple schemas are simultaneously active, facilitating the processing of the continuous and complex sensory information into meaning. Which level of specificity in novel stories successfully elicits resonance with the intended schema?

It is possible that lay intuitions about the right level of specificity may be quite accurate. Research by Baldassano and colleagues [65] investigated cortical event boundaries during narrative perception (specifically of movies and audio-narrations). The brain processes the continuous input in a hierarchy, with brain regions along this hierarchy segmenting information at increasingly long timescales (from sensory areas to multimodal conceptual areas). Baldassano et al. [65] demonstrated that event boundaries annotated by human observers mapped onto long neural events at the top of this hierarchy. In addition, event-related activity in these higher-order areas generalized across modalities (audio or movie) and between perception and recall [65–67]. Event-related activity in some of these higher-order areas also map (*e.g.*, the angular gyrus and posterior medial cortex) onto the corresponding schema’s temporal event structure [37]. To sum up, human intuitions about event boundaries and event-specificity in free recall map onto activity in areas associated with the activation of temporal event schemas. In addition, representations of event structure generalize across narrative modalities. Therefore, while it has not been tested that multiple versions of what we might intuitively identify as a Cinderella tale reliably activate the corresponding story schema, it is likely that the flexibility of these schemas in responding to relevant input might allow for some lenience. Most people’s intuitions about what constitutes a Cinderella tale may be quite effective. Future work should more directly test this question: Can we identify Cinderella-schema specific neural activity in the narrative perception of different Cinderella-type stories (and across different mediums)?

The studies reported here employ a novel story similarity measure. This measure was built for the purposes of tracking the degree of change across story retellings, and similarity across participants. While it is not suited for all story comparisons, we propose that this measure, as well as variations of this measure, may be useful in future memory and cultural research. Repeated reproduction designs (as was done here), chain reproduction designs—as in telephone game studies, as well as social network designs could benefit from more automatic tracking of mnemonic changes in more naturalistic recall. The Story Similarity measure offers a simple, and easily interpretable metric, while still affording a degree of automaticity in analysis. Our approach to measuring story dispersion may also provide easily visualizable insight into mnemonic convergence in networks over time. Given the rapid advances in NLP tools, this measure should only improve with time. In particular, this measure could be improved with: (1) More accurate means of marking event boundaries or accommodating texts of varying lengths [68]; (2) A more rigorous, human-validated threshold for determining whether text segments represent the same underlying events; and (3) Standardization of similarity values to allow for comparisons across studies and stories of varying lengths. Nevertheless, the underlying logic of this measure remains helpful in tracking changes in both content and sequence of narratives or episodic recollections. Parts of the measure may also be adapted to track: (1) which events or elements of a report are more likely to be remembered or forgotten, both within and across participants, (2) whether certain elements are more likely to remembered or forgotten *together*, or (3) which elements are more likely to be rearranged in recollections of an episode or narrative.

## Conclusion

In the three studies reported here, we employ novel natural language processing analyses to illustrate how a familiarly structured story possesses an evolutionary edge over incoherent and unfamiliar stories. All stories used here are new to participants, and yet we find that a story with a familiar structure not only survives better within individuals’ memory, it survives more consistently across people. Future work should explore why certain stories survive. Cross-cultural research would be critical to understanding: What about a story structure makes it a good, strong story? One that will be remembered and passed down through the generations within its cultural context, or passed along across cultural contexts. Are good stories those that teach us about our social worlds?

## Supporting information

S1 Appendix Familiar story structures possess an evolutionary edge in memory. (DOCX)
