# Supplementary material for: Familiar story structures possess an evolutionary edge in memory
Source: PLoS One. 2026 Mar 3;21(3):e0341671. doi: 10.1371/journal.pone.0341671 (PMC12956119; doi:10.1371/journal.pone.0341671)
Supplement: S1 Appendix — (DOCX) [file pone.0341671.s001.docx]

**S1 Appendix**

**Validating the Story Similarity Measure**

In addition to testing the story similarity measure against human ratings of similarity, we wanted to make sure that the measure is not simply picking up on differences in length between stories. Or at least, not more so than would a human rater. We found no significant correlations between the story similarity measure and either the word count (*r*(92)= -0.08, *p*=0.46 (ns) or sentence count difference (*r*(92)= -0.11, *p*=0.28 (ns)). Nor do we find any significant correlations between human ratings and either the word count (*r*(92)= -0.08, *p*=0.42 (ns)) or sentence count difference (*r*(92)= -0.05, *p*=0.63(ns)). In other words, story similarity, as calculated by the story similarity measure and as judged by human raters, is not determined by differences in story length.

**Study 1**

**Nonsense Story**

Sophia is a psychic. Her job is to talk with dead scientists from beyond the grave. Naturally, she is always careful to get permission from their closest living relatives. On an evening like any other, as she is working to set up a new contact, Sophia is invited to join a training program for an army of intergalactic peace keepers. She quickly realizes she must learn traditional forms of dance, and she goes to a legendary witch with many years of experience. The witch teaches her how to catch the most elusive of prey – the notorious double-edged sword. Sophia befriends the wild beast, and convinces it to search for treasure, hidden in an amusement park deep in the Amazonian forest. They travel together, practicing their speech for the fateful day, giving each other advice on the most persuasive changes in tone. Unfortunately, during a particularly eloquent turn of phrase, they slip in the mud and are separated. Each one spirals down a different path. Sophia finds herself locked in the pantry in the restaurant at the end of the world. She is not too worried, as she has food fit for a swarm of locusts. But, eventually, Sophia grows tired of the company of insects. They never seem to be able to tell her apart from other humans. Luckily, Sophia is able to drink from a magic waterfall that gives her a heightened sense of smell. She finds a mysterious set of bear paw prints and tries to follow them down a drain, swimming as fast as she can. Having trouble catching up, Sophia calls on a small spacecraft that she shares with her mother. The spacecraft has the temperament of a werewolf grumpily waking up from a nap, but knows how to step up when needed. Flying along, Sophia gets ambushed by a giant rat and almost eaten. Luckily, Sophia bumps into her good friend, a lawyer named Lesley, who happens to have good ties with the underground community. With their help, she is able to talk herself out of the situation and embarks on a new project to bolster the area’s local economy. Inspired, Sophia builds a robot replica of herself, capable of sharing her thoughts, but, luckily, free of any desire to replace the original.

*Word Count:* 378

**Results**

**Table 1**

*Similarity Across Retellings (Study 1)*

*Note.* This table shows the pairwise comparisons between the similarity of subsequent retellings and all following subsequent retellings.

**Table 2**

*Similarity to the Last Retelling (Study 1)*

*Note.* This table shows the pairwise comparisons between (1) the similarity of each retelling to the last retelling (produced on Day 5) and (2) the similarity of each subsequent retelling to the last retelling.

**Table 3**

*Similarity to the Initial Story (Study 1)*

*Note*. This table shows the pairwise comparisons between (1) the similarity of each retelling to the initial story and (2) the similarity of each subsequent retelling to the initial story.

**Study 2**

**Coherent Story (same as the Familiar Story in Study 3)**

Sara had grown up in one of the poorer neighborhoods of the city. She dreamed of attending college and pursuing a degree in physics, but she knew that it would be difficult. Nowadays, it was getting harder and harder to stand out amongst the pool of applicants, especially if you came from a school with fewer means. Her junior year, though, her whole high school was abuzz: One of the premier universities of the city was organizing a science fair, and all the students in the area would be invited to participate. Students with the top projects would be awarded a scholarship to their program. In an effort to be fair, the university had set two rules: One, all schools had to agree to the same budget for their students’ projects, and, two, the judging would be blind—judges would not be allowed to know who the students were or what school they were from. Sara was excited. For months, she worked hard on her project, putting in all her free time. The week leading up to the it, students’ families were getting ready for the science fair: Many had set up transport the day before—the university, where the fair was taking place, was a bit far. Unfortunately, Sara’s parents, who were often very busy at work, were not able to take her. Sara assured them that she could make her way there and back on her own, and they gave her enough for the train rides. Sara got there early to register and set up her project. She was given a card with an ID code to input to the fair’s website once the winners were announced. It was how the judges would identify the students. Sara, afraid of losing the card, recorded the code on her phone. During the fair, everyone was presenting from behind screens. It was a bit of an odd setup, but somehow people made it work. People were not allowed to access the internet while in the fair hall, to make sure that no one could look up answers to questions. The judges were also anonymous, going around amongst the crowd of attendees—faculty, college students, and visitors who had heard about the event. Sara had a wonderful time, and barely felt the hours go by. One conversation in particular had been especially stimulating: The person on the other side of the screen seemed to be really taking to her work, asking pointed questions that pushed Sara’s thinking further and further, in ways that surprised even her. The fair was nearing its end when she got an alert. The train she was supposed to take back, the last one that day, was out of service. If she wanted to get back home that evening, she would have to rush out at once to catch an earlier train. A bit frantic, she gathered up her things and ran out into the night, sprinting all the way to the station. She made it just in time. Finally taking a breath, she looked through her things. In a start, she realized that she had left one of her bags behind. It was the bag containing her phone and her wallet, with the ID card in it. A few days passed. Sara could not bring herself to check the fair’s website for the winners. She had gotten in touch with the fair organizers, to try and get her bag (and the code) back, explain her situation. Unfortunately, in all the uproar of the fair and the clean-up after, a lot of things had been misplaced. Sara tried to resign herself to what had happened, trying to convince herself that she hadn’t really lost anything. Her parents, who asked her about the fair several times only to get mumbled half-replies, decided to check the results themselves: The winning projects had all been announced, but one had remained unclaimed. The student had not been identified. Sara’s parents, recognizing their daughter’s work, shouted for her. Sara, barely believing the news, called the organizers again: Without the ID code, they could not be sure of her, but, luckily, one of the judges had had the foresight to come up with questions they were certain only the project creator could answer properly. They put her through to the judge: Sara recognized the voice of her pointed questioner on the other end of the line. With a renewed confidence, Sara answered the questions with ease. The judge recognized her too, and happily awarded Sara her prize. She had won the scholarship.

*Word Count:* 758

**Incoherent Story (scrambled version of the Coherent Story)**

Sara had grown up in one of the poorer neighborhoods of the city. She dreamed of attending college and pursuing a degree in physics, but she knew that it would be difficult. Sara was given a card with an ID code to input to a website once the winners were announced. It was how the judges would identify the students. Sara was excited. For months, she worked hard on her project, putting in all her free time. Sara, barely believing the news, called the organizers again: They put her through to the judge. Sara recognized the voice of her pointed questioner on the other end of the line. Sara tried to resign herself to what had happened, trying to convince herself that she hadn’t really lost anything. During the fair, everyone was presenting from behind screens. It was a bit of an odd setup, but somehow people made it work. People were not allowed to access the internet while in the fair hall, to make sure that no one could look up answers to questions. The week leading up to it, students’ families were getting ready for the science fair: Many had set up transport the day before—the university, where the fair was taking place, was a bit far. Unfortunately, Sara’s parents, who were often very busy at work, were not able to take her. With a renewed confidence, Sara answered the questions with ease. Nowadays, it was getting harder and harder to stand out amongst the pool of applicants, especially if you came from a school with fewer means. Without the ID code, they could not be sure of her, but, luckily, one of the judges had had the foresight to come up with questions they were certain only the project creator could answer properly. Students with the top projects would be awarded a scholarship to their program. In an effort to be fair, the university had set two rules: One, all schools had to agree to the same budget for their students’ projects, and, two, the judging would be blind—judges would not be allowed to know who the students were or what school they were from. Sara assured them that she could make her way there and back on her own, and they gave her enough for the train rides. She had won the scholarship. Her parents, who asked her about the fair several times only to get mumbled half-replies, decided to check the results themselves. Sara got there early to register and set up her project. The judge recognized her, and happily awarded Sara her prize. A bit frantic, she gathered up her things and ran out into the night, sprinting all the way to the station. She made it just in time.  Sara was afraid of losing the card, so she recorded the code on her phone. One conversation in particular had been especially stimulating: The person on the other side of the screen seemed to be really taking to her work, asking pointed questions that pushed Sara’s thinking further and further, in ways that surprised even her. The winning projects had all been announced, but one had remained unclaimed. The student had not been identified. The judges were also anonymous, going around amongst the crowd of attendees—faculty, college students, and visitors who had heard about the event. The fair was nearing its end when Sara got an alert. The train she was supposed to take back, the last one that day, was out of service. If she wanted to get back home that evening, she would have to rush out at once to catch an earlier train. Sara had a wonderful time, and barely felt the hours go by. Sara could not bring herself to check the fair’s website for the winners. Finally taking a breath, she looked through her things. In a start, she realized that she had left one of her bags behind. It was the bag containing her phone and her wallet, with the ID card in it. A few days passed. She had gotten in touch with the fair organizers, to try and get her bag (and the code) back, explain her situation. Unfortunately, in all the uproar of the fair and the clean-up after, a lot of things had been misplaced. Sara’s parents, recognizing their daughter’s work, shouted for her. Sara’s junior year, though, her whole high school was abuzz: One of the premier universities of the city was organizing a science fair, and all the students in the area would be invited to participate.

*Word Count:* 758

**Results**

**Table 4**

*Similarity Across Retellings (Study 2)*

*Note.* This table shows the pairwise comparisons between the similarity of subsequent retellings and all following subsequent retellings for both the Coherent and Incoherent condition.

**Table 5**

*Similarity to Last Retelling (Study 2)*

*Note.* This table shows the pairwise comparisons between (1) the similarity of each retelling to the last retelling and (2) the similarity of each subsequent retelling to the last retellings for both the Coherent and Incoherent conditions.

*Similarity to Initial Story (Study 2):*

We did not find a main effect of day on similarity to the initial story (Modification measure). Collapsing both story conditions together, we find *F*(4,216) = 1.780, *p*=0.134, η^2^*_G_* = 0.012. Sensitivity analyses (as recommended in Lakens (2022) and calculated using the R package *pwr*) indicated that effects of approximately η² ≥ 0.067 would be detectable with 90% power at α = .05. Smaller effects can therefore not be reliably detected given the current design.

Separately, we found no main effect of day for the Coherent Story, *F*(4,120)=1.477, *p*=0.213, η^2^*_G_* = 0.016. The sensitivity analysis indicated that effects of approximately η² ≥ 0.114 would be detectable with 90% power at α = .05. In addition, we found no main effect of day for the Incoherent Story, *F*(4,96)=0.914, *p*=0.459, η^2^*_G_* = 0.016. The sensitivity analysis indicated that effects of approximately η² ≥ 0.138 would be detectable with 90% power at α = .05. For both the Coherent and Incoherent story, smaller effects can therefore not be reliably detected.

**Table 6**

*Stabilization between Conditions: Similarity Across Retellings (Study 2)*

| Days | Coherent | | Incoherent | | *F* | *p.adj* | η^2^*_G_* | CI.lower | | CI.upper |
| --- | --- | --- | --- | --- | --- | --- | --- | --- | --- | --- |
|  | *M* | *SD* | *M* | *SD* |  |  |  |  |  |  |
| Initial to Day1 | 0.258 | 0.092 | 0.069 | 0.077 | 67.380 | *** | 0.555 | 0.144 | 0.236 | |
| Day1 to Day2 | 0.522 | 0.154 | 0.337 | 0.158 | 19.391 | *** | 0.264 | 0.100 | 0.268 | |
| Day2 to Day3 | 0.611 | 0.167 | 0.419 | 0.176 | 17.503 | *** | 0.245 | 0.100 | 0.285 | |
| Day3 to Day4 | 0.605 | 0.189 | 0.464 | 0.172 | 8.306 | * | 0.133 | 0.043 | 0.239 | |
| Day4 to Day5 | 0.638 | 0.193 | 0.515 | 0.145 | 6.875 | * | 0.113 | 0.029 | 0.216 | |

*p<0.05 **p<0.01 ***p<0.001

**Table 7**

*Consistency between Conditions: Similarity to Last Retelling (Study 2)*

| Days | Coherent | | Incoherent | | *F* | *p.adj* | η^2^*_G_* | CI.lower | | CI.upper |
| --- | --- | --- | --- | --- | --- | --- | --- | --- | --- | --- |
|  | *M* | *SD* | *M* | *SD* |  |  |  |  |  |  |
| Initial to Day5 | 0.221 | 0.086 | 0.051 | 0.055 | 72.767 | *** | 0.574 | 0.130 | 0.209 | |
| Day1 to Day5 | 0.471 | 0.167 | 0.321 | 0.150 | 12.184 | ** | 0.184 | 0.064 | 0.236 | |
| Day2 to Day5 | 0.549 | 0.151 | 0.416 | 0.120 | 12.912 | ** | 0.193 | 0.059 | 0.208 | |
| Day3 to Day5 | 0.620 | 0.159 | 0.466 | 0.162 | 12.137 | ** | 0.184 | 0.064 | 0.237 | |
| Day4 to Day5 | 0.638 | 0.193 | 0.515 | 0.145 | 6.875 | 0.055 | 0.113 | 0.029 | 0.216 | |

*p<0.05 **p<0.01 ***p<0.001

**Table 8**

*Modification between Conditions: Similarity to Initial Story (Study 2)*

| Days | Coherent | | Incoherent | | *F* | *p.adj* | η^2^*_G_* | CI.lower | | CI.upper |
| --- | --- | --- | --- | --- | --- | --- | --- | --- | --- | --- |
|  | *M* | *SD* | *M* | *SD* |  |  |  |  |  |  |
| Initial to Day1 | 0.258 | 0.093 | 0.069 | 0.077 | 67.380 | *** | 0.555 | 0.144 | 0.236 | |
| Initial to Day2 | 0.239 | 0.103 | 0.053 | 0.057 | 64.672 | *** | 0.545 | 0.139 | 0.232 | |
| Initial to Day3 | 0.243 | 0.109 | 0.047 | 0.051 | 68.511 | *** | 0.559 | 0.148 | 0.243 | |
| Initial to Day4 | 0.234 | 0.098 | 0.061 | 0.052 | 63.685 | *** | 0.541 | 0.130 | 0.217 | |
| Initial to Day5 | 0.221 | 0.086 | 0.051 | 0.055 | 72.767 | *** | 0.574 | 0.129 | 0.209 | |

*p<0.05 **p<0.01 ***p<0.001

**Accounting for the Sample Size Difference**

Given the difference in sample size between conditions (*N*=31 for Coherent, *N*=25 for Incoherent), we wanted to rule out the possibility that it may be responsible for our main effects. To address this, we replicated the condition analyses with different subsets of 25 participants in the Coherent condition against all 25 participants in the Incoherent condition. We iterated through ANOVAs with 1000 random subsets of 25.

*Stabilization Results:* We always found a main effect of Condition. The Coherent story was always more similar across retellings than the Incoherent story: All 1000 generated *p*-values were below 0.001, with the max value generated being *p*=0.00016. Generated *F* values for the main effect of Condition were between 16.73 and 42.65, with *M*=24.46 and *SD*=4.31.

*Consistency Results:* We always found a main effect of Condition. The Coherent story retellings were always more similar to the final retellings than the Incoherent story retellings: All 1000 generated *p*-values were below 0.001, with the max value generated being *p*=0.0004. Generated *F* values for the main effect of Condition were between 14.26 and 45.28, with *M*=21.54 and *SD*=4.86.

*Modification Results:* We always found a main effect of Condition. The initial Coherent story was always better preserved across retellings than the Incoherent story: All 1000 generated *p*-values were below 0.0001. Generated *F* values for the main effect of Condition were between 79.99 and 151.47, with *M*=100.58 and *SD*=10.44.

**Table 9**

*Story Dispersion (Study 2)*

| Days | Coherent SDD | Incoherent SDD | Difference (p-value) |
| --- | --- | --- | --- |
| Day 1 | 0.095 | 0.100 | 0.774 (ns) |
| Day 2 | 0.099 | 0.102 | 0.831 (ns) |
| Day 3 | 0.101 | 0.107 | 0.731 (ns) |
| Day 4 | 0.100 | 0.107 | 0.671 (ns) |
| Day 5 | 0.090 | 0.095 | 0.776 (ns) |

**Study 3**

**Familiar Story (same as the Coherent Story in Study 2)**

Sara had grown up in one of the poorer neighborhoods of the city. She dreamed of attending college and pursuing a degree in physics, but she knew that it would be difficult. Nowadays, it was getting harder and harder to stand out amongst the pool of applicants, especially if you came from a school with fewer means. Her junior year, though, her whole high school was abuzz: One of the premier universities of the city was organizing a science fair, and all the students in the area would be invited to participate. Students with the top projects would be awarded a scholarship to their program. In an effort to be fair, the university had set two rules: One, all schools had to agree to the same budget for their students’ projects, and, two, the judging would be blind—judges would not be allowed to know who the students were or what school they were from. Sara was excited. For months, she worked hard on her project, putting in all her free time. The week leading up to the it, students’ families were getting ready for the science fair: Many had set up transport the day before—the university, where the fair was taking place, was a bit far. Unfortunately, Sara’s parents, who were often very busy at work, were not able to take her. Sara assured them that she could make her way there and back on her own, and they gave her enough for the train rides. Sara got there early to register and set up her project. She was given a card with an ID code to input to the fair’s website once the winners were announced. It was how the judges would identify the students. Sara, afraid of losing the card, recorded the code on her phone. During the fair, everyone was presenting from behind screens. It was a bit of an odd setup, but somehow people made it work. People were not allowed to access the internet while in the fair hall, to make sure that no one could look up answers to questions. The judges were also anonymous, going around amongst the crowd of attendees—faculty, college students, and visitors who had heard about the event. Sara had a wonderful time, and barely felt the hours go by. One conversation in particular had been especially stimulating: The person on the other side of the screen seemed to be really taking to her work, asking pointed questions that pushed Sara’s thinking further and further, in ways that surprised even her. The fair was nearing its end when she got an alert. The train she was supposed to take back, the last one that day, was out of service. If she wanted to get back home that evening, she would have to rush out at once to catch an earlier train. A bit frantic, she gathered up her things and ran out into the night, sprinting all the way to the station. She made it just in time. Finally taking a breath, she looked through her things. In a start, she realized that she had left one of her bags behind. It was the bag containing her phone and her wallet, with the ID card in it. A few days passed. Sara could not bring herself to check the fair’s website for the winners. She had gotten in touch with the fair organizers, to try and get her bag (and the code) back, explain her situation. Unfortunately, in all the uproar of the fair and the clean-up after, a lot of things had been misplaced. Sara tried to resign herself to what had happened, trying to convince herself that she hadn’t really lost anything. Her parents, who asked her about the fair several times only to get mumbled half-replies, decided to check the results themselves: The winning projects had all been announced, but one had remained unclaimed. The student had not been identified. Sara’s parents, recognizing their daughter’s work, shouted for her. Sara, barely believing the news, called the organizers again: Without the ID code, they could not be sure of her, but, luckily, one of the judges had had the foresight to come up with questions they were certain only the project creator could answer properly. They put her through to the judge: Sara recognized the voice of her pointed questioner on the other end of the line. With a renewed confidence, Sara answered the questions with ease. The judge recognized her too, and happily awarded Sara her prize. She had won the scholarship.

*Word Count:* 758

**Unfamiliar Story**

Sara had grown up in one of the poorer neighborhoods of the city. She dreamed of attending college and pursuing a degree in physics, though she knew that it would be difficult. It was getting harder and harder to stand out amongst the pool of applicants, especially if you came from a school with fewer means. Her senior year, though, her whole high school was abuzz: Her hard work had been recognized, and she had won a scholarship to one of the premier universities in the area. After starting college, Sara decided to help organize a science fair the university was holding for students in the area. She wanted to help other students get a chance like the one she had gotten. Students with the top projects would be awarded a scholarship to their program. She convinced the organizing committee that, to be fair, they should set two rules: One, all schools had to agree to the same budget for their students’ projects, and, two, the judging would be blind—judges would not be allowed to know who the students were or what school they were from. Sara was excited. For months, she worked hard to set up the fair, putting in all her free time. The week leading up to the science fair, Sara visited her neighborhood and was glad to see that many students in the area were getting ready for it. She helped set up transport to the university, where the fair was taking place. The university was a bit far and she knew that some parents, who were often very busy with work, might not be able to get their children there. The neighborhood wanted to support their students, though, and many families offered their help—including Sara’s own. During the fair, everyone was presenting from behind screens. It was a bit of an odd setup, but somehow people made it work. People were not allowed to access the internet while in the fair hall, to make sure that no one could look up answers to questions. The judges were also anonymous, going around amongst the crowd of attendees—faculty, college students, and visitors who had heard about the event. Sara had a wonderful time, and barely felt the hours go by. She enjoyed walking around the hall, seeing students’ work, listening to them present. Once in a while, she would stop to ask a few pointed questions. She was often pleased by the students’ enthusiasm and the quality of their thinking. The fair was nearing its end when she got an alert. She had almost forgotten about an evening class she had to attend. A bit sad to be leaving early, Sara quickly gathered up her things and went out into the night, rushing to class. She made it just in time. Finally taking a breath, she looked through her things. In a start, she realized that she had left one of her bags behind. It was the bag containing her phone and her wallet. A few days passed. Sara had not been able to find her missing bag. In all the uproar of the fair and the clean-up after, a lot of things had been misplaced. Sara was a little frustrated with herself, but could not really bring herself to be mad. All in all, the science fair had been a success. There was only one hiccup. The winning projects had all been announced, but one had remained unclaimed. The student had not been identified. Sara’s parents, who had come to really care about the science fair and the opportunity it could mean for students in the neighborhood, insisted that Sara find out who it was. They were convinced it was a student from her old school. Sara, remembering the project, realized there might be a way to identify the student. She asked teachers from participating schools to present their classes with a series of questions she was certain only the project creator would be able to answer properly. She was right, and her parents had been right. The student belonged to Sara’s old school. They had been hesitant to come forward, scared about what accepting the scholarship would mean—leaving their family, and the life they knew, behind. Sara, understanding their fear, shared her own experience with the student. She tried to show them that they could go on to college, and still remain active in the neighborhood they had grown up in. Moving forward did not mean abandoning their community. The student came forward to claim their scholarship.

*Word Count:* 756

**Results**

**Table 10**

*Similarity Across Retellings (Study 3)*

*Note.* This table shows the pairwise comparisons between the similarity of subsequent retellings and all following subsequent retellings for both the Familiar and Unfamiliar condition.

**Table 11**

*Similarity to Last Retelling (Study 3)*

*Note.* This table shows the pairwise comparisons between (1) the similarity of each retelling to the last retelling and (2) the similarity of each subsequent retelling to the last retellings for both the Familiar and Unfamiliar conditions.

*Similarity to Initial Story (Study 3):*

We did not find a main effect of day on similarity to the initial story (the Modification measure). Collapsing across story conditions, we find *F*(4,212) = 2.399, *p*=0.051, η^2^*_G_* = 0.018. Sensitivity analyses indicated that effects of approximately η² ≥ 0.068 would be detectable with 90% power at α = .05. Smaller effects cannot be reliably detected given the current design.

**Table 12**

*Stabilization between Conditions: Similarity Across Days (Study 3)*

| Days | Familiar | | Unfamiliar | | *F* | *p.adj* | η^2^*_G_* | CI.lower | | CI.upper |
| --- | --- | --- | --- | --- | --- | --- | --- | --- | --- | --- |
|  | *M* | *SD* | *M* | *SD* |  |  |  |  |  |  |
| Initial to Day1 | 0.260 | 0.055 | 0.165 | 0.093 | 20.820 | *** | 0.282 | 0.053 | 0.136 | |
| Day1 to Day2 | 0.460 | 0.159 | 0.392 | 0.126 | 3.080 | ns | 0.055 | -0.010 | 0.146 | |
| Day2 to Day3 | 0.505 | 0.141 | 0.485 | 0.188 | 0.196 | ns | 0.004 | -0.070 | 0.110 | |
| Day3 to Day4 | 0.535 | 0.155 | 0.539 | 0.164 | 0.008 | ns | 0.000 | -0.091 | 0.083 | |
| Day4 to Day5 | 0.569 | 0.134 | 0.539 | 0.129 | 0.740 | ns | 0.014 | -0.041 | 0.102 | |

*p<0.05 **p<0.01 ***p<0.001

*Note.* We did not find a main effect of story condition on the similarity across days of retelling for Study 3, *F*(1,53) = 1.950, *p*=0.17, η^2^*_G_* = 0.023. A sensitivity analysis indicated that effects of approximately η² ≥ 0.165 would be detectable with 90% power at α = .05. Effects below this threshold can therefore not be reliably detected.

**Table 13**

*Consistency between Conditions: Similarity to Last Retelling (Study 3)*

| Days | Familiar | | Unfamiliar | | *F* | *p.adj* | η^2^*_G_* | CI.lower | | CI.upper |
| --- | --- | --- | --- | --- | --- | --- | --- | --- | --- | --- |
|  | *M* | *SD* | *M* | *SD* |  |  |  |  |  |  |
| Initial to Day5 | 0.231 | 0.059 | 0.147 | 0.064 | 25.156 | *** | 0.322 | 0.050 | 0.117 | |
| Day1 to Day5 | 0.478 | 0.150 | 0.338 | 0.159 | 10.971 | 0.01 | 0.171 | 0.055 | 0.222 | |
| Day2 to Day5 | 0.490 | 0.169 | 0.450 | 0.187 | 0.721 | ns | 0.013 | -0.056 | 0.137 | |
| Day3 to Day5 | 0.560 | 0.117 | 0.500 | 0.188 | 2.176 | ns | 0.039 | -0.023 | 0.147 | |
| Day4 to Day5 | 0.569 | 0.134 | 0.539 | 0.129 | 0.740 | ns | 0.014 | -0.041 | 0.102 | |

*p<0.05 **p<0.01 ***p<0.001

**Table 14**

*Modification between Conditions: Similarity to Initial Story (Study 3)*

| Days | Familiar | | Unfamiliar | | *F* | *p.adj* | η^2^*_G_* | CI.lower | | CI.upper |
| --- | --- | --- | --- | --- | --- | --- | --- | --- | --- | --- |
|  | *M* | *SD* | *M* | *SD* |  |  |  |  |  |  |
| Initial to Day1 | 0.260 | 0.055 | 0.165 | 0.093 | 20.820 | *** | 0.282 | 0.053 | 0.136 | |
| Initial to Day2 | 0.241 | 0.087 | 0.153 | 0.077 | 15.420 | ** | 0.225 | 0.042 | 0.131 | |
| Initial to Day3 | 0.215 | 0.090 | 0.180 | 0.080 | 2.432 | ns | 0.044 | -0.010 | 0.082 | |
| Initial to Day4 | 0.228 | 0.083 | 0.136 | 0.073 | 19.323 | *** | 0.267 | 0.050 | 0.134 | |
| Initial to Day5 | 0.231 | 0.059 | 0.147 | 0.064 | 25.156 | *** | 0.322 | 0.050 | 0.118 | |

*p<0.05 **p<0.01 ***p<0.001

**Table 15**

*Story Dispersion (Study 3)*

|  | Familiar SDD | Unfamiliar SDD | Difference (p-value) |
| --- | --- | --- | --- |
| Day 1 | 0.081 | 0.103 | 0.185 (ns) |
| Day 2 | 0.083 | 0.092 | 0.518 (ns) |
| Day 3 | 0.083 | 0.105 | 0.149 (ns) |
| Day 4 | 0.076 | 0.112 | 0.033* |
| Day 5 | 0.070 | 0.104 | 0.046* |

* p<0.05

**Google Ngram**

This graph, made using Google Ngram, shows the relative number of mentions of popular fairytale title n-grams between 1800 and 2022.

**
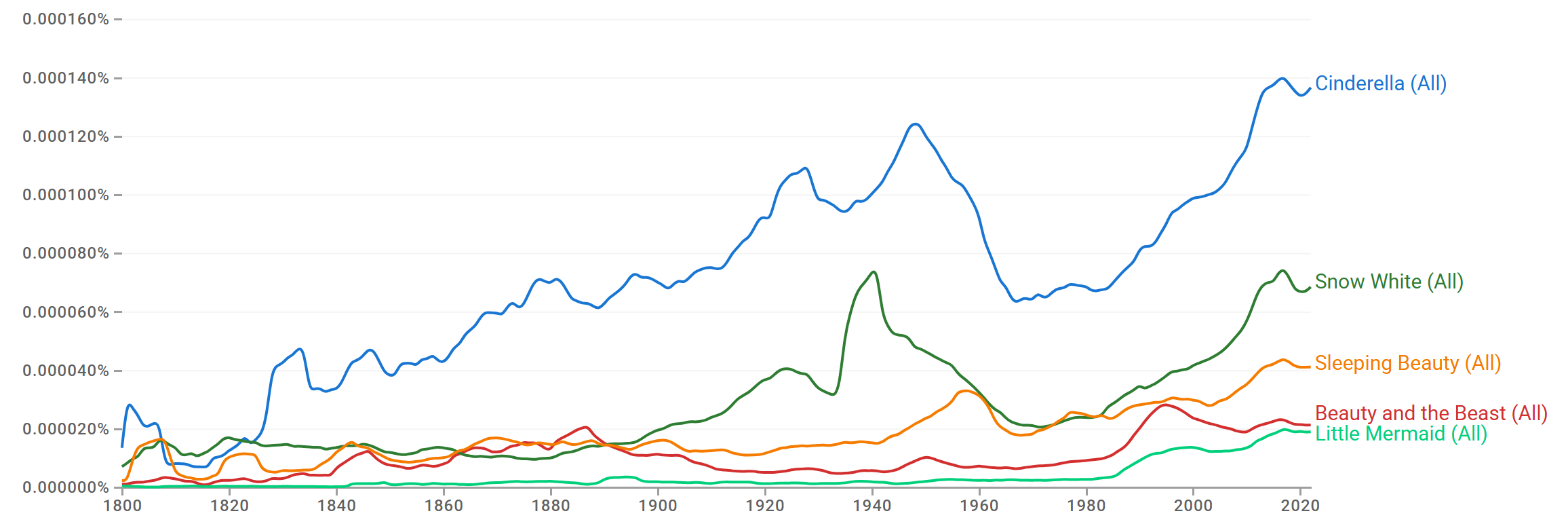
**
